# Structural characterization of recombinant humanized type III collagen cold gel and its effects on the biological functions of HaCaT cells

**DOI:** 10.3389/fmed.2026.1749460

**Published:** 2026-02-10

**Authors:** Yizhe Wang, Sibin Kuang, Yuhui Fan, Danfeng Li, Ling Li, Jian Wang, Wei Bian

**Affiliations:** 1 Shanxi Medical University, Taiyuan, China; 2 Shanxi Key Laboratory of Functional Proteins, Shanxi Jinbo Bio-Pharmaceutical Co., Ltd., Taiyuan, China

**Keywords:** C3Gel, cryo-electron microscopy, keratinocytes, thermal stability, triple helix, wound healing

## Abstract

Recombinant humanized collagen has been widely used in the biomedical field. This study focuses on the characterization of a novel recombinant humanized type III collagen cold gel (C3Gel). To verify the structural integrity of C3Gel and explore its *in vitro* cytocompatibility and potential biological applications, a multi-technique approach covering composition, structure, thermal properties, and morphology was employed to perform comprehensive structural analysis. In addition, *in vitro* biological functional assessments were conducted using cell-based assays. The results demonstrated that the molecular weight and structural sequence of C3Gel were consistent with theoretical expectations. Under low-temperature conditions, C3Gel self-crosslinked into a porous network structure and exhibited favorable cytocompatibility. It enhanced the microenvironment of the extracellular matrix (ECM) and offered substantial structural support to HaCaT cells, which in turn facilitated their adhesion, proliferation, and migration. These findings suggest that C3Gel holds promise as a novel functional biomedical material, providing a scientific basis for further research and application of this recombinant collagen.

## 1 Introduction

Collagen constitutes a major structural protein in mammals and is fundamental to the structural integrity of diverse human tissues including skin, bone, tendon, and muscle. As a principal component of the extracellular matrix in connective tissues, it holds significant promise for biomedical applications (1). However, conventional collagen—typically sourced from animal tissues—faces limitations such as complex extraction protocols, immunogenicity risks, potential viral contamination, and low water solubility. Recombinant collagen technology has consequently emerged as a strategic solution to these challenges and is increasingly regarded as an ideal biomaterial for biomedical use (2).

Commercial recombinant human collagen products are typically supplied as lyophilized fibers that can reconstitute with solutions. Although convenient, such collagen solutions provide limited spatial support. This limitation is effectively addressed by utilizing collagen in a gel state. When prepared and stored under appropriate low-temperature conditions, collagen gels undergo a process of molecular self-assembly, ultimately forming a distinctive three-dimensional network. This architecture closely mimics the natural extracellular matrix of human tissues, featuring an interconnected porous structure that promotes cell adhesion, migration, and proliferation. Consequently, these gels serve as an excellent scaffold to support tissue repair and regeneration (3).

Characterizing recombinant collagen is essential for both its research and practical application. Through systematic characterization, its structural features, biological activity, and physicochemical properties can be fully elucidated, thereby providing a theoretical foundation for its use in tissue engineering, drug delivery, wound healing, and related fields (4). In recent years, numerous studies have employed a variety of analytical methods to support the structural identification and functional validation of recombinant collagen. For example, as early as 1998, Myers et al. used amino acid analysis to confirm the primary structure of recombinant collagen, establishing a basis for subsequent research into its effects on murine arthritis (5). In another study focused on the preparation and identification of recombinant collagen, Yan et al. have combined ultraviolet-visible spectroscopy (UV-Vis), circular dichroism (CD), and scanning electron microscopy (SEM) to verify its triple-helical structure and visualize its fibrous morphology (6) Furthermore, Fourier transform infrared spectroscopy (FTIR) is widely applied to identify molecular structures and distinguish between collagen types (7). Despite these advances, comprehensive structural characterization and biofunctional assessment of recombinant collagen in its gel form remain underexplored, highlighting a key gap for future investigation.

This study presents the first systematic investigation into the multi-scale structural and functional properties of a commercial, low-temperature self-assembling recombinant humanized type III collagen gel (C3Gel). By integrating multiple analytical techniques—including peptide mapping using liquid chromatography-mass spectrometry (LC-MS), protein binary structural analysis using circular dichroism (CD), and microstructural visualization with scanning and cryo-electron microscopy (SEM and Cryo-EM)—we established a complete structural profile from primary sequence to ultrastructure. This multi-technique approach significantly enhances the comprehensiveness and reliability of the characterization. Assessments of thermal stability and *in vitro* biofunctionality further demonstrate the hydrogel’s exceptional biomimicry of native extracellular matrix microenvironments. Together these findings provide a robust theoretical foundation and experimental validation for the application of C3Gel in biomedical fields such as tissue engineering and drug delivery.

## 2 Materials, instruments, and methods

### 2.1 Materials

The C3Gel used in this work was supplied by Shanxi Jinbo Biological Medicine Co., Ltd., at a concentration of 16 mg/mL. The reference material of bovine type I collagen (bCol-I) was acquired from the National Institute for Food and Drug Control (China). Acetonitrile and formic acid were procured from Sigma-Aldrich and Tokyo Chemical Industry Co., Ltd., respectively. Ultrapure water was generated in-house using a Millipore water purification apparatus. Physiological saline was sourced from Shijiazhuang No. 4 Pharmaceutical Co., Ltd. The HaCaT cell line, an immortalized human keratinocyte model, was obtained from Guangdong Biosea Biotechnology Co., Ltd. DMEM high-glucose medium and fetal bovine serum (FBS) were purchased from Thermo Fisher Scientific Inc., while PBS and D-PBS buffers were provided by Beijing Solarbio Science & Technology Co., Ltd. Bovine serum albumin (BSA) and the penicillin-streptomycin solution came from Sigma-Aldrich and Shanghai Beyotime Biotechnology Co., Ltd., respectively. Cell viability assessments were carried out with a Cell Counting Kit-8 (CCK-8) (Nanjing Vazyme Biotech Co., Ltd.).

### 2.2 Instruments

The following instrument models were used in the structural characterization section: A ViscoQC 300 cone-plate viscometer (Anton Paar, Austria); a high-performance liquid chromatography system Nexera LC-40D (SHIMADZU, Japan) and a X500B QTOF high-resolution mass spectrometer (SCIEX, Canada) for molecular weight analysis; an ultra-performance liquid chromatography system ACQUITY UPLC I-Class (Waters, United States) and a high-resolution mass spectrometer Xevo G2-XS Qtof (Waters, United States) for peptide mapping analysis; Chirascan PLUS V100 CD spectrometer (Applied Photophysics, United Kingdom); a Helios G4 UC scanning electron microscope (ThermoFisher, United States), a cryo-electron microscope Glacios 200 kV (ThermoFisher, United States; and a micro differential scanning calorimeter Microcal PEAQ-DSC (Malvern Panalytical, United Kingdom).

The following instruments were used in the cell experiments: A Roche Cedex HiRes automated cell counter (Roche, Switzerland), a microplate reader Synergy H1 (BioTek, United States), an inverted fluorescence microscope EVOS M5000 (Thermo Fisher Invitrogen, United States), and a Heracell V10s 250i CO_2_ incubator (Thermo Scientific, United States).

### 2.3 Methods of structural characterization

#### 2.3.1 Dynamic viscosity

One milliliter of C3Gel was placed in the test cup of a cone-plate viscometer (Anton Paar, ViscoQC 300). Using rotor CP52, the measurement was conducted under the following conditions: rotational speed of 30 rpm, temperature of 5.0°C, density set to 1 g/cm^3^, and a step width of 10 s. The dynamic viscosity was continuously recorded over a period of 200 s. The resulting data were plotted as a dynamic viscosity–time curve using Origin software.

#### 2.3.2 Molecular weight determination by LC-MS

##### 2.3.2.1 Sample preparation

0.1 mL of C3Gel (16 mg/mL) was diluted with 1.5 mL of purified water to achieve a final concentration of 1 mg/mL.

##### 2.3.2.2 Chromatographic conditions

Separation was performed employing a Biozen 1.8 μm dSEC-2 column (200 Å, 150 × 4.6 mm) with an injection volume of 5 μL. The column temperature was consistently held at 40 °C. Mobile phase A comprised 0.1% formic acid in aqueous solution, while mobile phase B consisted of 0.1% formic acid in acetonitrile. The flow rate was set to 0.2 mL/min, and detection was achieved using UV absorption at 214 nm. Isocratic elution was implemented utilizing a blend of 65% mobile phase A and 35% mobile phase B over a 20-min duration (8).

##### 2.3.2.3 Mass spectrometric conditions

Electrospray ionization (ESI) was used as the ion source and the positive ion mode was selected as the detection method with a scan range of 500–4,000 m/z.

##### 2.3.2.4 Data processing

The acquired data were deconvoluted using SCIEX software to generate the molecular weight profile and subsequently imported into Origin for further analysis and graphical representation.

#### 2.3.3 Peptide mapping analysis (primary structure and amino acid sequence analysis)

##### 2.3.3.1 Sample preparation

A 0.1 mL aliquot of C3Gel (16 mg/mL) was diluted with 1.5 mL of purified water to obtain a final concentration of 1 mg/mL. Then, 100 μg of the diluted C3Gel was transferred to an ultrafiltration device and diluted with 150 mM Tris buffer (pH 7.8). The sample was subsequently subjected to reduction and alkylation using dithiothreitol (DTT) and iodoacetamide, respectively. After buffer exchange into 150 mM Tris (pH 7.8), Lysyl Endopeptidase (Lys-C) was added and the sample was incubated at 37 °C for 60 min. The reaction was terminated by adding formic acid (FA), and the resulting digest was injected into the ultra-performance liquid chromatography system for analysis.

##### 2.3.3.2 Chromatographic conditions

Chromatographic separation was carried out using a reversed-phase column (Waters, ACQUITY UPLC Peptide BEH C18 Column, 2.1 mm × 150 mm, 1.7 μm). Mobile phase A was composed of 0.1% formic acid in aqueous solution, while mobile phase B contained 0.1% formic acid in acetonitrile. The gradient elution conditions are detailed in Table 1. A constant flow rate of 0.3 mL/min was maintained throughout the 40-min analysis.

**TABLE 1:** Chromatographic gradient conditions.

Time (min)	A(%)	B(%)
0.00	100	6
3.00	100	6
30.00	77	13
30.10	0	100
33.00	0	100
33.10	100	0
40.00	100	0

##### 2.3.3.3 Mass spectrometric conditions

The detection was performed in positive ion MSE mode with precursor ion scanning over a mass range of 300–2,000 m/z.

##### 2.3.3.4 Data processing

The raw data were processed using UNIFI software (Waters) with the parameters specified in Table 2.

**TABLE 2:** Software analysis parameters.

Item	Value
Enzyme	Lys-C
Fixed modification	Carbamidomethyl (C)
Variable modifications	Oxidation MW, Deamidation NQ
M/Z tolerance	±10 ppm
Fragment match tolerance	±10 ppm

#### 2.3.4 Circular dichroism spectroscopy

C3Gel was diluted with physiological saline to concentrations of 4, 2, 1, and 0.5 mg/mL. For each concentration, 200 μL of the solution was immediately transferred to a cuvette with a 1 mm path length and subjected to spectral scanning using a circular dichroism spectrometer. The scanning protocol was configured with the following parameters: wavelength range 190–260 nm, bandwidth 1.0 nm, step size 1.0 nm, time-per-point 1 s, and scan rate 50 nm/min. A 0.5 mg/mL solution of bCol-I served as the reference standard.

The spectrum of physiological saline was recorded as the baseline. All acquired spectra were processed using the software Pro-Data Viewer for baseline subtraction and smoothing. The processed data was then exported to Origin for further graphical refinement.

#### 2.3.5 Scanning electron microscopy analysis

A 10 μL aliquot of C3Gel was pipetted onto a sample carrier and rapidly plunged into liquid nitrogen slush for approximately 30 s. The cryo-fixed sample was then transferred under vacuum using a Quorum cryo-transfer device to a pre-cooled preparation chamber maintained at –140 °C. Under cryogenic and vacuum conditions, the sample was randomly fractured. Subsequently, it was subjected to freeze sublimation at –70 °C for 10 min, followed by sputter coating with platinum to enhance conductivity.

The prepared sample was transferred via a cryo-transfer shuttle onto the cold stage of a Helios G4 UC cryo-scanning electron microscope, where it was observed at –140 °C. Imaging was performed under an accelerating voltage of 10 kV and a beam current of 13 pA. Micrographs were acquired at appropriate magnifications.

#### 2.3.6 Cryo-electron microscopy analysis

Under conditions of 2–8°C, C3Gel was diluted with physiological saline to a concentration of 0.4 mg/mL, ensuring that the gel state was maintained throughout the dilution process. The mixture was gently inverted for homogenization. A 5 μL aliquot was aspirated and applied onto a grid using a Thermo Fisher Vitrobot. The blot force was set to 0 and the blot time was 3 s. A Quantifoil 1.2/1.3 300 mesh Au grid was used for sample support.

The prepared sample was subsequently plunge-frozen in liquid ethane. Imaging was performed using a Glacios cryo-electron microscope operated at 200 kV, with a magnification of 120,000 × and a total electron dose of 50 e^–^/Å^2^.

#### 2.3.7 Differential scanning calorimetry thermal stability analysis

A 0.1 mL volume of C3Gel was diluted with 1.5 mL of physiological saline to achieve a protein concentration of 1 mg/mL for the determination of its thermal denaturation temperature (Tm). After acquiring a baseline scan using physiological saline, which showed minimal response and good reproducibility, 250 μL of the diluted C3Gel was loaded for measurement. The temperature was scanned from 15 to 100°C at a heating rate of 90°C/h. All measurements were performed under a nitrogen atmosphere.

### 2.4 Evaluation of cellular biological functions of C3Gel

#### 2.4.1 Cytotoxicity

C3Gel was subjected to dilution with physiological saline and subsequently combined in a 1:1 ratio with DMEM complete medium supplemented with 10% FBS and 1% penicillin-streptomycin, yielding final C3Gel concentrations of 0, 0.25, 0.5, 1, 2, 4, and 8 mg/mL. The group containing 0 mg/mL C3Gel was designated as the control.

A cellular suspension at a density of 1 × 10^5^ cells/mL was introduced into a 96-well plate at a volume of 100 μL per well and maintained under culture conditions (37 °C, 5% CO_2_) for 24 h. The supernatant was carefully aspirated, followed by two washing steps with PBS. Thereafter, 100 μL of the previously prepared C3Gel solutions, diluted in DMEM complete medium, were administered to each well. After an additional 24-h incubation period, the solutions were discarded, and the wells underwent two further washes with PBS. Next, 100 μL of CCK-8 solution, diluted to 5% in basal DMEM medium, was introduced into each well. Following a 3-h incubation, the absorbance was measured at 450 nm using a microplate reader.

Cell viability in the control group was established as 100%. The viability of cells exposed to varying concentrations of C3Gel was calculated as a percentage relative to the control group. Statistical significance was assessed through one-way analysis of variance (ANOVA).

#### 2.4.2 Cell adhesion

C3Gel was diluted with D-PBS buffer to concentrations of 0.25, 0.5, 1, and 2 mg/mL for experimental use. A 1 mg/mL solution of bovine type I collagen served as the positive control (PC), while D-PBS buffer was used as the negative control (NC). A volume of 100 μL of each experimental group (diluted in D-PBS), PC, and NC was added into individual wells of a 96-well plate. Following covering, the plate was incubated overnight at 4°C.

After incubation, the supernatant was discarded, and each well was blocked with 100 μL of 1% BSA in D-PBS at 37 °C for 60 min. The solution was then removed, and the wells were gently washed twice with D-PBS. HaCaT cells were plated at a density of 2 × 10^6^ cells/mL in 100 μL aliquots per well. The cells were then cultured for 120 min under standard conditions (37°C, 5% CO_2_). Following incubation, the supernatant was carefully aspirated using a multichannel pipette, and the wells were washed twice with 100 μL D-PBS. Subsequently, 100 μL of DMEM basal medium containing 5% CCK-8 solution was added to each well, and the plate was incubated for 2 h. A microplate reader was utilized to measure the absorbance at 450 nm.

Cell viability in the NC group was defined as 100%. The viability of cells in all other groups was calculated as a percentage relative to the NC group. Statistical significance was evaluated using one-way analysis of variance (ANOVA).

#### 2.4.3 Cell migration

Cells were resuspended in medium supplemented with 10% FBS and plated into a 12-well plate with a final density of 3 × 10^5^ cells/mL (1 mL per well), using three replicate wells per group. To minimize the impact of cell proliferation on scratch healing and ensure HaCaT cells remain in optimal condition to achieve significant healing effects at the 24-h mark, we performed starvation treatment and subsequent experimental procedures using DMEM medium supplemented with 1% FBS after 24 h of cell attachment. Upon reaching over 85% confluence, a linear scratch was introduced employing a standard 200 μL pipette tip. Culture medium solutions for different experimental groups were prepared in advance. The negative control group (NC) was prepared using DMEM starvation medium containing 1% FBS. The experimental groups were established using C3Gel solutions with final concentrations of 0.125, 0.5, and 1 mg/mL prepared in the same medium. The formulated solutions for every experimental group, along with the NC group, were subsequently administered. Scratch images were acquired at intervals of 0, 12, 24, and 36 h employing an inverted fluorescence microscope under 10 × magnification. The scratch width was quantified utilizing Photoshop software, enabling computation of the cellular migration rate. For comparative analysis, statistical significance was determined by two-way ANOVA.

All data from cellular assays were processed and graphically represented with GraphPad Prism software, version 8. Quantitative findings are reported as mean ± standard deviation (SD). Statistical significance was assessed based on the following criteria: *, *P* ≤ 0.05; **, *P* ≤ 0.01; ***, *P* ≤ 0.001.

## 3 Results

### 3.1 Structure characterization

#### 3.1.1 Dynamic viscosity

**FIGURE 1:**
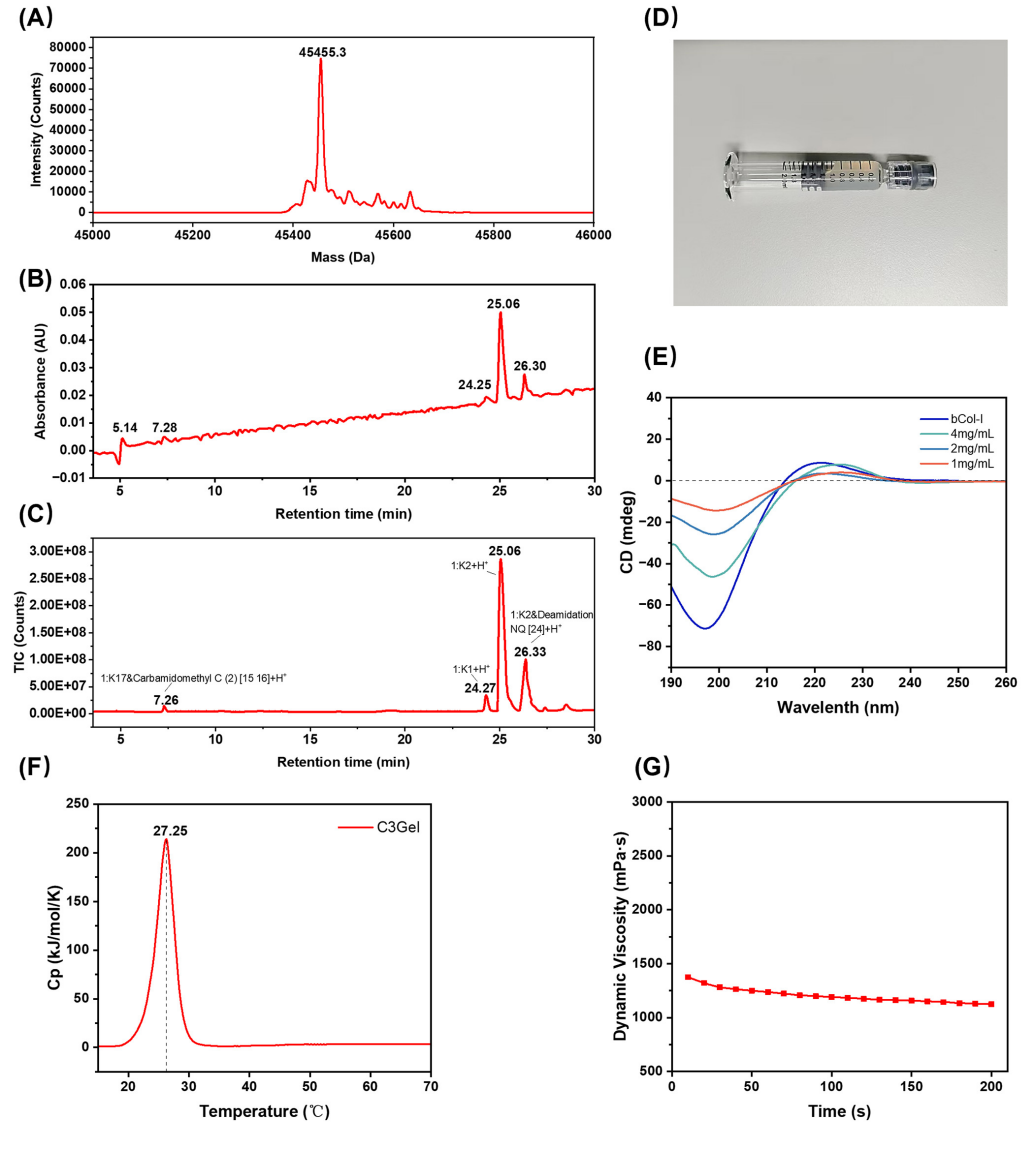
**(A)** Mass spectrum of C3Gel showing molecular weight. **(B)** Liquid chromatography profile of the peptide map. **(C)** Total ion current (TIC) chromatogram of the peptide map. **(D)** Macroscopic appearance of C3Gel. **(E)** Circular dichroism spectra of C3Gel at different concentrations and bCol-I reference. **(F)** DSC thermogram of C3Gel. **(G)** Dynamic viscosity–time curve of C3Gel.

C3Gel exhibited a white, semi-transparent gel-like appearance (Figure 1D). The formation of this appearance arises from the primary sequence of C3Gel being rich in specific repetitive functional segments Gly-X-Y. This enables multiple single strands to twist into a triple helix structure through hydrogen bonds, salt bridges, and hydrophobic interactions. Furthermore, based on the presence of Cys residues in the primary sequence, it forms interchain covalent disulfide bonds, aggregating the triple helix structures into oligomers. The numerous charged side chains on the surface of these oligomers then undergo lateral alignment into fibrous bundles through electrostatic attraction and hydrogen bonding, ultimately cross-linking into a networked gel. Under the specified parameters, the measured shear rate was 59.94/s. The dynamic viscosity over a period of 200 s is shown in Figure 1G, with an average dynamic viscosity of 1202.4 mPa⋅s. This value, being greater than 1,000 mPa⋅s, indicates that the protein possesses favorable mechanical strength and can maintain a relatively stable gel state (3).

#### 3.1.2 Molecular weight analysis

C3Gel is composed of 489 amino acids with a theoretical repeating sequence of (GERGAPGFRGPAGPNG IPGEKGPAGERGAP)16 + GAPGPCCGG and a theoretical molecular weight of 45,454.2 Da. Analysis of the LC-MS spectrum (Figure 1A) showed that the measured molecular weight was 45,455.3 Da, which is consistent with the theoretical value.

#### 3.1.3 Peptide mapping and peptide sequence analysis

Lys-C is an enzymatic protein belonging to the serine protease family, which selectively hydrolyzes peptide bonds at the C-terminal end of lysine residues and digests large proteins into smaller peptide fragments. After enzymatic digestion of C3Gel with Lys-C, followed by LC-MS separation and scanning, the UV chromatogram (Figure 1B) and total ion current (TIC) mass spectrum (Figure 1C) of the resulting digest are shown. Peptide matching and identification were performed using UNIFI software (Waters), which revealed 100% coverage of the theoretical peptide sequence, confirming that the detected sequence is consistent with the theoretical one.

#### 3.1.4 CD

The CD results of proteins reflect their tertiary structure, and this technique is commonly used to analyze the secondary structure of biological protein molecules. To further assess the structural integrity of C3Gel, we subjected it to analysis using a CD spectrometer. As shown in Figure 1E, a positive absorption peak appeared above zero within the 200–230 nm range, accompanied by a distinct negative peak below zero at 190–200 nm. And this result applies to all tested concentrations. These features are consistent with those of the reference bCol-I, which are typical of a triple-helical tertiary structure (9). As the concentration went up, we observed a corresponding increase in peak intensity. It indicates a higher content of the triple-helical structure. Collectively, these data verify the preserved structural integrity of C3Gel.

#### 3.1.5 SEM

**FIGURE 2:**
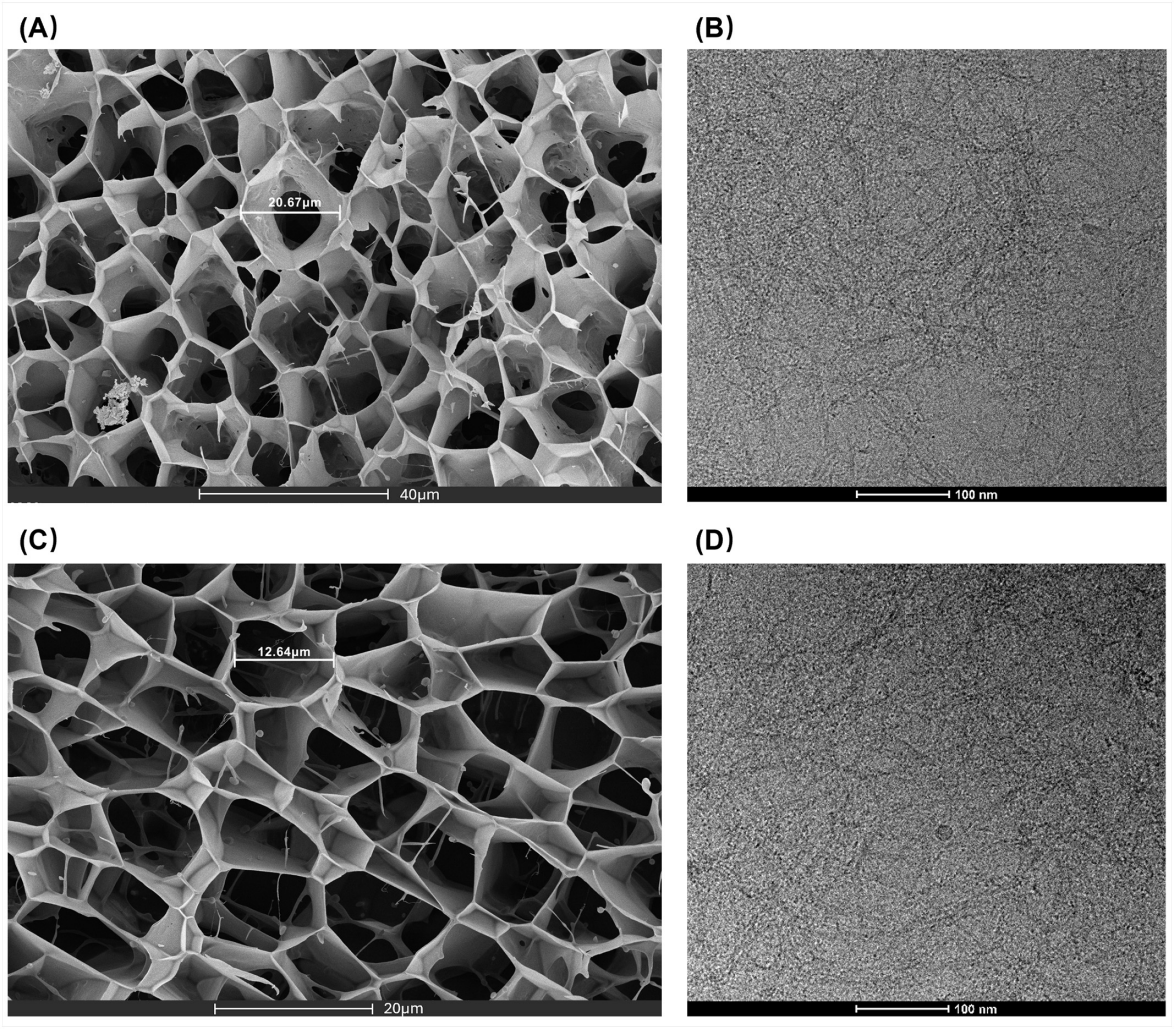
**(A**,**C)** SEM images of C3Gel. **(B**,**D)** Cryo-EM images of C3Gel.

SEM imaging (Figure 2) reveals that C3Gel successfully formed a uniform, three-dimensional network through self-crosslinking. This structure contains tightly arranged micropores measuring 10–30 μm in size. Notably, this range is comparable to the scale of skin cells like keratinocytes and fibroblasts (10, 11). This pore morphology mimics the physical characteristics of the natural ECM. It can create a biomimetic structure that is highly conducive to cell adhesion and function. The high porosity not only provides critical support for cell attachment and migration (12) but also enables efficient permeation of body fluids and nutrients (13), which is essential for tissue engineering applications.

#### 3.1.6 Cryo-EM

The Cryo-EM image (Figure 2) reveals the morphology of C3Gel at a more detailed nanoscale, demonstrating that C3Gel self-assembles into a fibrous structure under cryogenic conditions. The measured fiber length ranges between 30 and 45 nm. This dimensional scale is closely linked to the mechanical properties of collagen fibers (14), cellular mechanotransduction, and tissue repair (15). Moreover, it suggests a potential key role in promoting interfacial integration within bone tissue (16).

#### 3.1.7 DSC Thermal stability analysis

Thermal analysis is commonly used to study physical changes—such as polymorphic transitions, melting, and evaporation—or chemical changes—including thermal decomposition and oxidation—that occur in substances upon heating, along with accompanying alterations in temperature, energy, or weight. DSC is a widely used method for assessing the thermal stability of proteins (17). It records the heat flow difference between a sample and a reference during a controlled temperature increase, enabling the determination of the material’s Tm.

The Tm refers to the melting temperature of a crystal or the denaturation temperature of a biomacromolecule. In the context of protein unfolding thermodynamics, it represents the thermal denaturation temperature at which half of the ordered triple-helical structure unwinds into a disordered random coil. For collagen-based materials, this transition reflects the thermal stability of the gel network and is observed as an endothermic peak in the DSC scan (18). A higher Tm indicates better thermal stability of the collagen protein.

Analysis of the DSC curve of C3Gel (Figure 1F) showed that as the ambient temperature increased, C3Gel began to absorb heat around 20 °C, with a distinct endothermic peak appearing at 27.25 °C. This temperature corresponds to the Tm of C3Gel, representing the midpoint of the triple helix-to-coil transition.

### 3.2 *In vitro* study: effects on cellular behavior

#### 3.2.1 Cytotoxicity

The cytotoxicity assay was conducted to evaluate whether C3Gel at various concentrations exerts any toxic effects on HaCaT cells, thereby assessing its *in vitro* biosafety. After 24 h of treatment, the group without C3Gel was used as the control, with its cell viability set as 100%. The viability of other groups was compared to that of this control.

**FIGURE 3:**
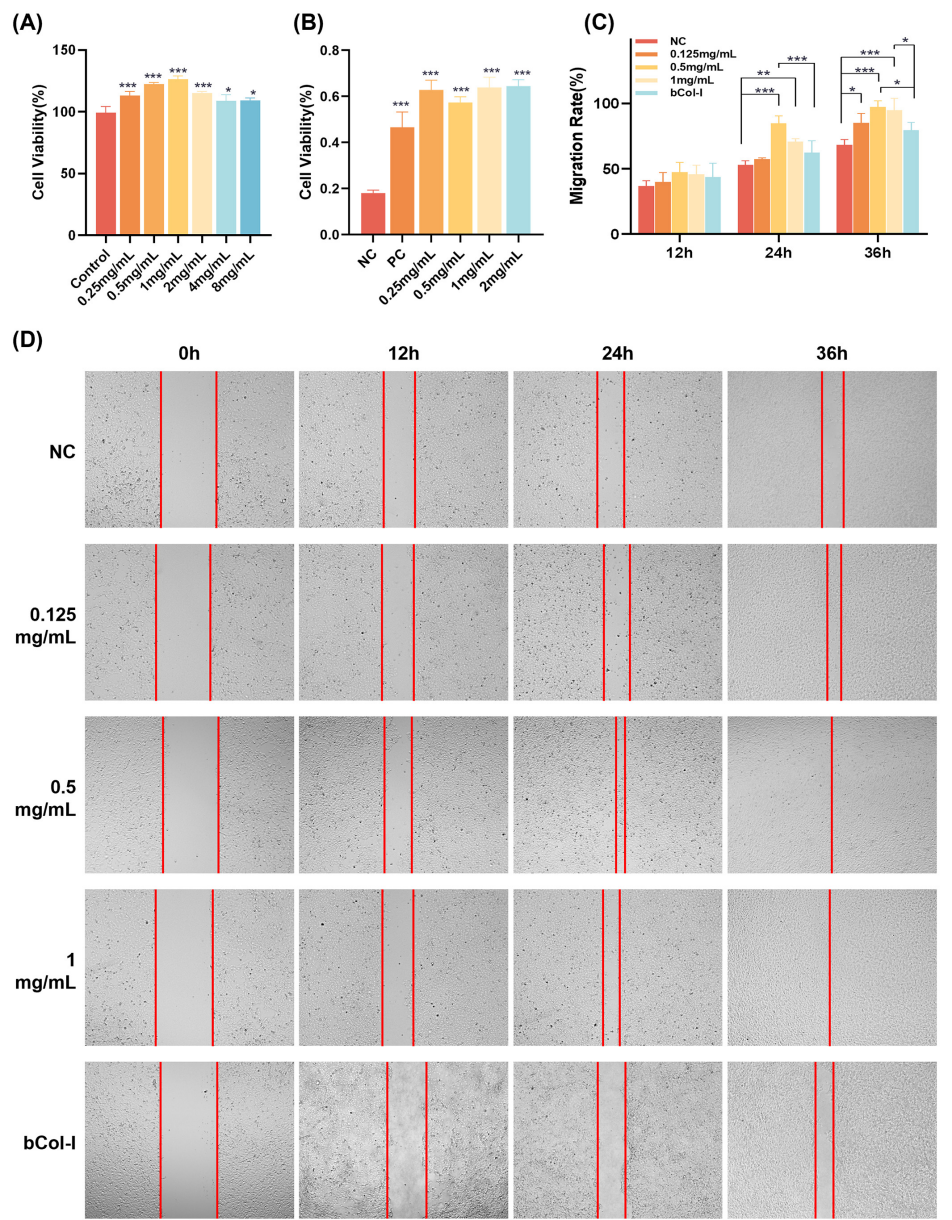
**(A)** Bar graph of cytotoxicity assay of collagen at different concentrations. **(B)** Bar graph showing cell adhesion analysis of collagen at different concentrations. **(C)** Quantitative analysis of scratch wound closure *in vitro* for experimental and control groups. **(D)** Microscopic images of cell scratch regions at different treatment time points (*P ≤ 0.05, **P ≤ 0.01, ***P ≤ 0.001).

The results of the cytotoxicity assay (Figure 3A) demonstrated that C3Gel exhibits excellent cytocompatibility. Furthermore, all concentration groups showed statistically significant differences compared to the control, with higher cell viability, indicating a pro-proliferative effect of C3Gel. This effect exhibited concentration dependence: within the range of 0.25–1 mg/mL, cell viability showed an increasing trend, with highly significant differences (*P* ≤ 0.001) compared to the control. In contrast, at concentrations ranging from 1 to 8 mg/mL, the promotive effect on cell proliferation gradually decreased. Based on these findings, subsequent experiments were conducted using a concentration of 1 mg/mL.

#### 3.2.2 Cell adhesion

Cell adhesion, as the initial stage of cell-material interaction, serves as a critical prerequisite for anchoring, spreading, and subsequent cell proliferation on material surfaces. This process significantly influences cellular physiological activities and determines whether cells can stably survive and perform their biological functions in a new environment (19).

As shown in Figure 3B, the viability of adhered HaCaT cells was evaluated after 120 min of incubation in 96-well plates coated with different concentrations of C3Gel. The results demonstrated that within the concentration range of 0.25–2 mg/mL, the cell adhesion rates showed significant differences compared to the D-PBS negative control group, indicating that C3Gel enhances cell adhesion.

#### 3.2.3 Cell migration

To examine the potential impact of recombinant collagen C3Gel on keratinocyte migration within a composite wound healing context, HaCaT cells were incubated in a culture medium containing C3Gel, followed by observation of cellular movement within the scratched region. As illustrated in Figures 3C,D, exposure to medium incorporating 0.125, 0.5, and 1 mg/mL C3Gel over periods of 12, 24, and 36 h resulted in varying degrees of increase in HaCaT cell migration into the wound area across all groups relative to the NC group. By 24 h, the 0.5 mg/mL group demonstrated the most effective promotion of scratch healing (*P* ≤ 0.001). At 36 h, the 0.5 and 1 mg/ml groups exhibited near-complete wound closure, with both concentrations demonstrating significantly higher rates of healing compared to the bCol-I group (*P* ≤ 0.05). These findings reveal that C3Gel especially in the concentration 0.5 mg/mL potently stimulates cellular migration and enhances the wound closure process in the scratch assay model.

## 4 Discussion

The results of peptide mapping and peptide sequence analysis in this study confirmed that the amino acid sequence of C3Gel is consistent with the theoretical design. Its molecular weight, determined by LC-MS, was 45,454.3 Da. In comparison, natural type III collagen has a molecular weight of approximately 417 kDa (20), making C3Gel approximately one-tenth the molecular weight of natural type III collagen, which may facilitate better absorption by human tissues.

The triple-helical structure of collagen consists of three α-chains. Procollagen is initially synthesized and undergoes intracellular post-translational modifications before self-assembling into the triple-helical structure, which is characterized by repeating Gly-X-Y (G-X-Y) motifs (21). The presence of these triple-helical units enables the formation of interchain disulfide bonds via cysteine residues, leading their aggregation into oligomers. Driven by electrostatic attraction and hydrogen bonding among their charged side chains, these oligomers then align laterally to form fiber bundles, which eventually resulting in a cross-link gel network. C3Gel’s sequence obeys this collagen pattern, so the triple helix must first be verified. CD showed positive and negative peaks within the expected wavelength range, confirming this configuration. It fosters greater thermal stability and mechanical strength and possesses the capacity to modulate cell surface receptor binding and subsequent signal transduction, which may directly influences cellular function and response (22).

SEM and Cryo-EM analyses confirmed that C3Gel formed a porous fibrous network structure by self-assembly and molecular self-crosslinking, which is based on the formation of the triple helix structure. Similar to the natural extracellular matrix (ECM) environment, this network exhibits excellent toughness and elasticity and provides mechanical support for cells (15). This makes it ideal material for tissue engineering applications. *In vitro* experiments including cell adhesion, cytotoxicity, and migration assays with HaCaT cells demonstrated C3Gel’s favorable cytocompatibility, strong cell-adhesive properties, and effectiveness effectively promoting scratch wound healing. These findings indicate its considerable potential for wound-healing therapies, tissue engineering, and aesthetic medicine. However, given that wound healing involves the coordinated action of multiple cell types—such as keratinocytes, fibroblasts, and endothelial cells—and that this study was limited to HaCaT cells *in vitro*, the practical therapeutic applicability of C3Gel requires further validation through subsequent *in vivo* studies.

The thermal denaturation temperature (Tm) of C3Gel, determined by differential scanning calorimetry (DSC) to be 27.25°C, reveals its thermal stability for storage and application. This indicates that it is stable at room temperature (25°C) but may denature at human body temperature (37°C). To improve its thermal stability, different strategies could be considered such as structural modification, cross-linking agents, or composite matrix materials. In a study by a research team at the College of Food Science, Northwest University, the triple-helical structure of fish collagen was stabilized using heat shock protein 47 (Hsp47), enabling non-covalent assembly and raising the denaturation temperature of the collagen to 37.7°C (23). In another research inspired by pangolin scales, Xiu Shi et al. employed a stable film (Col/DA/ZnO/BW) from collagen, diphenylaldehyde-terminated polyethylene glycol, ZnO nanoparticles, and black wolfberry anthocyanin, achieving a thermal stability of up to 71.17°C through covalent and hydrogen bonding (24). Furthermore, analysis of 1,200 collagen samples has established a positive correlation between the proportion of hydrophobic residues and melting temperatures (25). Computational tools, such as the neural network database ThermoMPNN, also can be used as a promising tool for the predictive design of thermally stable collagen variants (26). Collectively, these studies provide a valuable foundation for guiding efforts to improve the thermal stability of C3Gel.

## 5 Conclusion

This study investigated a novel recombinant humanized type III collagen-based cryogel (C3Gel) through a series of structural characterization techniques, confirming its structural integrity and specificity. Compared with existing studies, we utilized SEM and Cryo-EM analyses to determine the fiber length and pore structure diameter of C3Gel. These dimensions are close to certain biological length scales in the human body, contributing to its superior biocompatibility. And we used DSC analysis to investigate its thermal stability, revealing a prominent advantage for storage and transportation while also identifying a potential application limitation. *In vitro* cell experiments demonstrated that C3Gel exhibits favorable cell adhesion and cytocompatibility toward HaCaT cells, and it effectively promotes scratch wound healing. All the results suggest that C3Gel holds potential for biomedical and tissue engineering applications, providing an important experimental basis for the use of recombinant collagen in tissue engineering, medical aesthetics, and other biomedical fields.

## Data Availability

Data are available from the corresponding author upon reasonable request.
